# Genotoxicity of Silver Nanoparticles in *Vicia faba:* A Pilot Study on the Environmental Monitoring of Nanoparticles

**DOI:** 10.3390/ijerph9051649

**Published:** 2012-05-04

**Authors:** Anita K. Patlolla, Ashley Berry, LaBethani May, Paul B. Tchounwou

**Affiliations:** 1 Molecular Toxicology Research Laboratory, NIH-RCMI Center for Environmental Health, College of Science Engineering and Technology, Jackson State University, Jackson, MS 39217, USA; Email: ashleyberry@gmail.com (A.B.); labeth@yahoo.com (L.M.); paul.b.tchounwou@jsums.edu (P.B.T.); 2 Department of Biology, Jackson State University, Jackson, MS 39217, USA; 3 Murray High School Student-SEPA Program, Jackson State University, Jackson, MS 39217, USA

**Keywords:** silver nanoparticles, chromosomal aberrations, mitotic index, *Vicia faba*, genotoxicity, micronucleus

## Abstract

The use of silver nanoparticles (AgNPs) in commercial products has increased significantly in recent years. Although there have been some attempts to determine the toxic effects of AgNPs in mammalian and human cell-lines, there is little information on plants which play a vital role in ecosystems. The study reports the use of *Vicia faba* root-tip meristem to investigate the genotoxicity of AgNPs under modified GENE-TOX test conditions. The root tip cells of *V. faba* were treated with four different concentrations of engineered AgNPs dispersion to study toxicological endpoints such as mitotic index (MI), chromosomal aberrations (CA) and micronucleus induction (MN). For each concentration, five sets of microscopy observations were carried out. The results demonstrated that AgNPs exposure significantly increased (p < 0.05) the number of chromosomal aberrations, micronuclei, and decreased the MI in exposed groups compared to control. From this study we infer that AgNPs might have penetrated the plant system and may have impaired mitosis causing CA and MN. The results of this study demonstrate that AgNPs are genotoxic to plant cells. Since plant assays have been integrated as a genotoxicity component in risk assessment for detection of environmental mutagens, they should be given full consideration when evaluating the overall toxicological impact of the nanoparticles in the environment.

## 1. Introduction

Nanomaterials are part of an industrial revolution to develop lightweight but strong materials for a variety of purposes [1]. Due to the novel physical and chemical properties of nanoscale materials, nanomaterials have been used to create new consumer products as well as applications for life sciences and biotechnology. Chemically, the nanoparticles are very diverse. It is estimated that of all the nanomaterials used in consumer products, silver nanoparticles (AgNPs) currently have the highest degree of commercialization [2], so they are more likely to be exposed to humans and to the environment at large. The toxic effects of nanoparticles have been evaluated in a variety of studies; however the potential health and environmental impacts on plants have yet to be thoroughly examined. Their uniquely small size and large surface area is a key indicator of toxicity which allows them to translocate when inhaled [3]. Most recently, nanomaterials such as single- and multi-walled nanotubes, nanofibers, fullerene derivatives, quantum dots, and metal oxide nanoparticles have received much attention due to their toxicity on human cells, bacteria, and rodents [4,5,6,7,8,9,10,11]. With increasing interest in its potential toxicity, the adverse effects of engineered nanomaterials are intensively being investigated. To date, the studies that report on toxic effects of AgNPs either *in vivo* [12,13,14] or *in vitro* [15,16,17,18] further provide data indicating adverse health effects of cells exposed to AgNPs. AgNPs have also been shown to be genotoxic in plant cells [19]. Moreover, the toxicity of AgNPs has been observed to be mediated through oxidative stress or the generation of reactive oxygen species (ROS) as revealed by several studies [20,21]. Studies on potential toxicity of nanoparticles to ecological terrestrial test species are still lacking [22]. The studies on both positive and negative effects of nanoparticles on higher plants are very few. Lu *et al*. [23] showed that nanoscale SiO_2_ and TiO_2_ enhanced nitrate reductase activity in soybean, and apparently hastened its germination and growth. Several studies reported that Nano-TiO_2_ promoted photosynthesis and nitrogen metabolism, and improved growth of spinach [24,25,26,27].

Exposure to nanoparticles can occur via water, food, cosmetics, drugs, and drug delivery devices, and can lead to a wide variety of toxicological effects [14]. Silver nanoparticles (AgNPs) have been rapidly employed in the manufacturing of many products such as healthcare items, room-sprays, pipelines, and washing machines due to its long-standing antibacterial properties [28,29]. It has been termed as a broad-spectrum biocide due to its ability to target a wide array of bacteria [30]. Silver impregnated catheters and wound dressings are used in therapeutic applications. In spite of the wide usage of AgNP in wound dressings, which can cause easy entry into the cells, very few reports on the toxicity of AgNPs are available. Several recently published reports state that despite the many promises of AgNPs, there are many unknown risks which have not been properly assessed prior to their high industrialized usage. Silver (Ag) is classified as an environmental hazard by the EPA because it is more toxic to aquatic plants and animals than any other metal except for mercury. Even if a nanoparticle itself is not especially toxic, silver nanoparticles increase the effectiveness of delivering toxic silver ions to locations where they can cause toxicity. In the near future there is a risk of enhanced bioavailability of the nanoparticles in the environment [13].

The mitotic root meristems of *Vicia faba* (broad beans) have been the pioneer cytogenetic materials for the detection of genotoxicity study of environmental pollutants. Based upon USEPA Gene-Tox Program chromosome aberration frequencies in the root-tips of *V. faba* have been used as indicators of genotoxicity. Plant assays have been integrated as a genotoxicity component in risk assessment for detection of environmental mutagens because of the simple, quick, inexpensive, efficient and reliable characters. The *V. faba* root tip chromosomal aberration assay is an established plant bioassay validated by the International Programme on Chemical Safety (IPCS, WHO) and the United Nations Environment Programme (UNEP) as an efficient and standard test for the chemical screening and *in situ* monitoring for genotoxicity of environmental substances. *V. faba* has been used for evaluating chromosomal aberrations since the 1920s [31,32,33,34]. 

Although AgNPs have been the subject of important toxicological research, there exists a lack of appropriate plant model for genotoxicity assessment. There is also a scarcity of scientific data describing the dose-response relationship with respect to their cytogenetic toxicity in plant systems. The reports from few previous studies have advanced our knowledge of toxicological impact of several types of nanomaterials. There are still many unresolved issues and challenges concerning the biological effects of nanoparticles. Therefore, the present study is designed to investigate clastogenic/genotoxic impacts of silver nanoparticles on *V. faba*.

## 2. Materials and Methods

### 2.1. Nanoparticles

**Figure 1:**
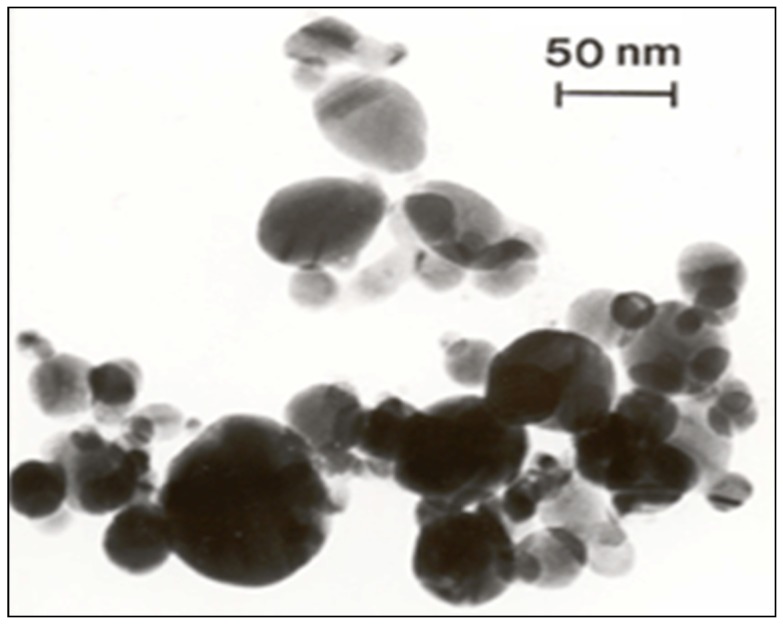
Transmission electron microscope image of silver nanoparticles.

Silver nanoparticles (AgNPs, Figure 1) were obtained from Ocean Nanotech LLC. (Fayetteville, AK, USA). The physical characteristics of the particles according to manufacturers’ data are 60 nm diameter size, 99.5% purity (trace metal basis), 400.0 m^2^/g surface area and 10.59 g/mL density.

### 2.2. Test System and Treatment

The Ag-NPs were suspended directly in deionized water (DI-water) and dispersed by ultrasonic vibration (100 W, 30 KHz) for 30 min to produce four different concentrations at 12.5 mg/L, 25 mg/L, 50 mg/L and 100 mg/L.

### 2.3. Plant as Test Material-Vicia faba

Several plant bioassays have been used for detecting mutagens and clastogens for the past 40 years. As a cytogenetic, material *V. faba* has the advantage of having six pairs of relatively large chromosomes that is excellent for assessing chromosomal aberrations. The *V. faba* root tip chromosomal aberration assay is an established plant bioassay by IPCS, WHO because of the simple, quick, inexpensive, efficient and reliable characters which can be utilized for *in situ* evaluation of the biological hazards of environmental pollution.

#### 2.3.1. Storage of Seeds:

The *V. faba* (Broad beans) seeds are preferably stored in a refrigerator with a temperature of 4 °C.

#### 2.3.2. Growing of Lateral Roots from Seeds

*V.* *faba* (broad bean) seeds were purchased from Carolina Biologicals (Burlington, NC, USA). Before use, seeds were disinfected by a short immersion (3–10 min) in a 5% sodium hypochlorite solution, followed by rinsing thoroughly with 3–4 changes of distilled water. The *V. faba* seeds were soaked in de-chloride tap water at 25 °C for 24 hour, and were then allowed to germinate between two layers of moistened cotton cloth in the dark for 3–5 days. When the primary root extended to 2–3 cm in length, the seed coat was peeled and the primary root tip was cut off (2 mm) in order to stimulate the emergence of secondary or lateral roots. The seedlings were suspended on water tanks and aerated constantly. The conditions for seedling growth were as follows 16 h light/8 h dark, 25 °C and light intensity of 5,000–10,000 Lux. The water in the tank was kept fresh by changing every day. The water tank was well aerated and maintained constant temperature (25 °C) during growth period. After 4 days in the water tank, seedlings with lateral roots of 1–2 cm were used in the assay.

#### 2.3.3. Chromosomal Aberration Assay

Four different concentrations of AgNPs (12.5, 25, 50 and 100 mg/L) and a control were used in *V. faba* chromosomal assay. The treatments with the test chemical (AgNPs) were carried out at for 4 h in the dark, in vials containing at least 25 mL of the treatment solution. Each concentration had five replications. After the treatment the seedlings were kept in distilled water for 24 h as recovery period. The temperature during the growth period of the seedlings, as well as during treatment and recovery, was maintained at 25 °C. Three hours prior to fixation the treated and the control root-tips were treated with 0.05% colchicine. 

#### 2.3.4. Fixation and Staining of Root Tips

Roots tips were fixed in freshly prepared fixative containing three parts methanol and one part glacial acetic acid and kept at 4 °C until later use. For preparing the root tips smears, they were removed from the refrigerator and transferred to room temperature in distilled water for 5 minutes. The root tips were then hydrolyzed in 1 N HCl at 60 °C for 6–7 minutes. After hydrolysis, the root tips were thoroughly washed with water several times and then stained with feulgen stain. When staining was completed, which took 45–60 minutes, the root-tips were transferred to clean slides and the dark stained tips containing the meristem were separated from the rest of the roots. Squash preparations were produced in 45% acetic acid.

#### 2.3.5. Scoring of Slides

In the *V. faba* chromosome aberration assay, slides were scored for chromatid and chromosome aberrations only in metaphase. Five hundred metaphases per root-tip were screened to a total of 2,500 metaphases for each treatment and control to obtain the total number of chromosomal aberrations. The mitotic indices were obtained by counting the number of mitotic cells in 1,000 cells/root-tip to a total of 5,000 cells/treatment and control using an Olympus microscope. The mitotic index was calculated as the ratio of the number of dividing cells to the total number of cells, multiplied by 100. The aberrations scored were chromatid breaks, isochromatid breaks, chromatid gaps, isochromatid gaps dicentrics, rings and lagging chromosomes. 

#### 2.3.6. Micronucleus Test

The procedure by Ma [32] was followed with slight modifications for this study. Seeds of *V. faba* were removed from storage and cultured at 28 °C with distilled water. Seedlings with lateral roots of *V. faba* at about 1–2 cm in length were collected for this experiment. The lateral roots were treated with four different concentrations (12.5, 25, 50 and 100 mg/L) and a control for 6 h at 28 °C, they were then transferred into distilled water for 44 h recovery time. The root tips were cut and fixed in a mixture of methanol and acetic acid (3:1, v/v). Before being squashed under a cover slip, the samples were hydrolyzed with 1N HCL at 70 °C for 7–8 min, stained with feulgen reagent for 2 h, and slides were made permanent. A total of 300 cells were analyzed, which were isolated from 3 different root tips (300 cells per root tip) for each sample. Micronuclei which localized inside the cell wall and in the cytoplasmic area surrounding the main nucleus with a diameter not exceeding one-third of the main nucleus were counted.

#### 2.3.7. Statistical Analysis

Statistical analysis was performed with SAS 9.1 software for Windows XP. Data was presented as Means ± SDs. One-way analysis of variance (ANOVA) with p-values less than 0.05 were considered as statistically significant.

## 3. Results

### 3.1. Nanomaterial Characterization

**Figure 2:**
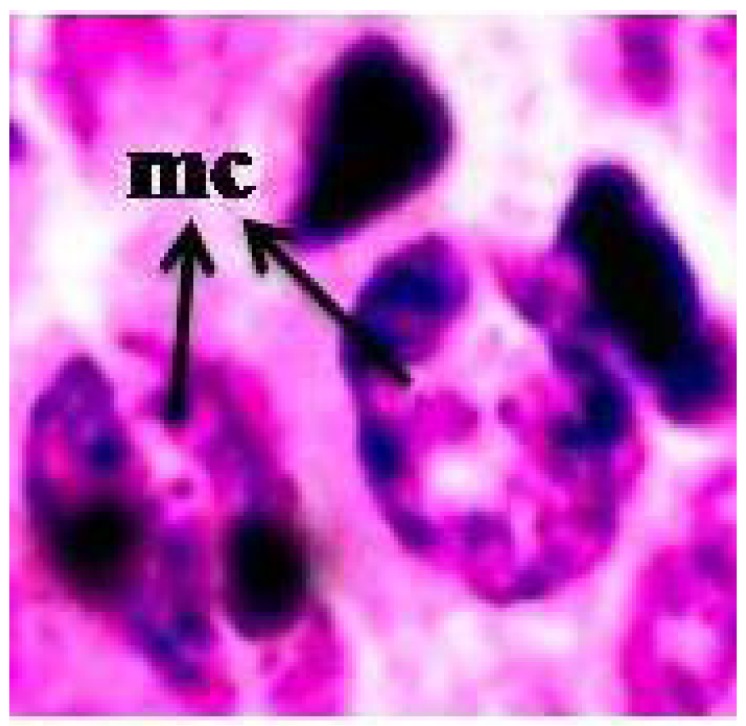
A histochemical stained (feulgen Stain) meristematic root-tip cell of *Vicia faba* showing silver nanoparticle inside viewed under BXI-40 Olympus Microscope (1,000×), mc = meristematic cell.

Nanoparticles were characterized by TEM with respect to morphology, diameter, tendency of aggregation and cellular distribution. AgNPs were mainly spherical shaped (Figure 1). Observing 300 particles, a mean longitudinal diameter of 63 ± 41 nm (mean ± SE) and a mean lateral diameter of 36 ± 21 nm (mean ± SE) could be measured. Hence, particle sizes matched the declarations of their commercial supplier (60 nm). The uptake of Ag NPs by the *V. faba* meristematic root tip cells is characterized by histochemical method (feulgen staining) and viewed under BXI-40 Olympus microscope (1,000×) is shown in Figure 2. The release of Ag^+^ ions is demonstrated in Table 1.

**Table 1:** Mean release of Ag^+^ ions (mg/L) to the DI-water: n = not measurable.

Concentration (mg/L)	Release of Ag^+^ ion:	
	Exposure to Ag	Exposure to Ag NPs
Control	n	n
12.5	1.2	1.2
25	30.4	49.2
50	60.5	121.8
100	228.6	403.9

To understand the state of dispersion of the particles when placed into deionized water (DI-water), the AgNPs sample was analyzed by dynamic light scattering (DLS). The results from DLS showed agglomeration of Ag-NPs more than its primary size, and the zeta potential value of AgNPs was shown to be -33.2 mV. A solution is considered stable if the zeta potential value is more negative than -30 mV or more positive than +30 mV. 

### 3.2. Mitotic Index

**Figure 3:**
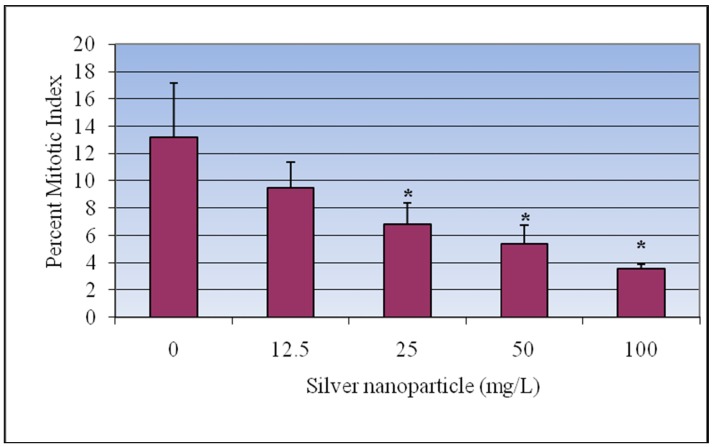
Effect of silver nanoparticles on the mitotic index in root-tip meristem of *Vicia faba*. Each experiment was done in triplicate. Data represents mean ± SD. Statistical significance (p < 0.05) is depicted as (*).

The mitotic index was used to determine the rate of cell division. The slides prepared for the assessment of chromosomal aberrations were used for calculating the mitotic index. It was found that the mitotic index significantly decreased as the Ag-NPs doses increased. Mitotic indices of 13.2 ± 3.98%, 9.48 ± 1.86%, 6.8 ± 1.57%, 5.42 ± 1.36%, and 3.56 ± 0.36% were recorded for 0, 12.5, 25, 50 and 100 mg/L of Ag-NPs respectively (Figure 3).

### 3.3. Chromosomal Aberrations

**Figure 4:**
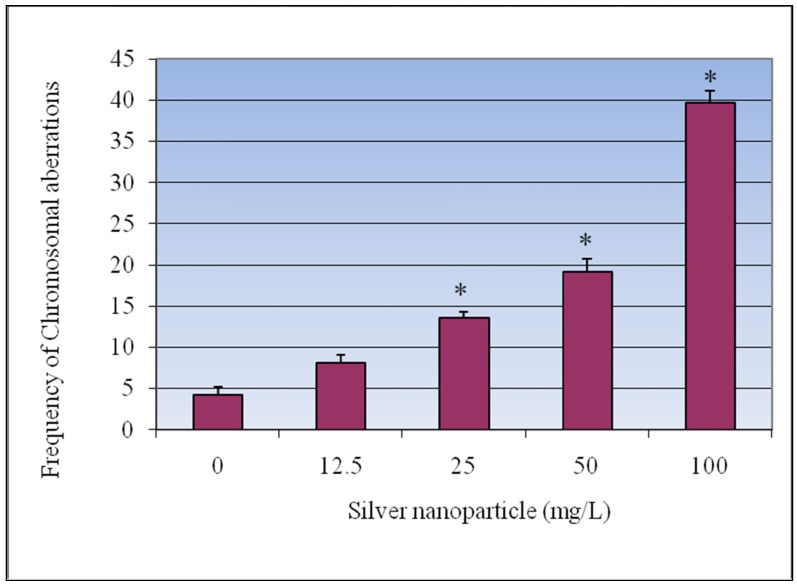
Effect of silver nanoparticles on the frequency of chromosomal aberrations in root-tip meristem of *Vicia faba*. Each experiment was done in triplicate. Data represents mean ± SD. Statistical significance (p < 0.05) is depicted as (*).

**Figure 5:**
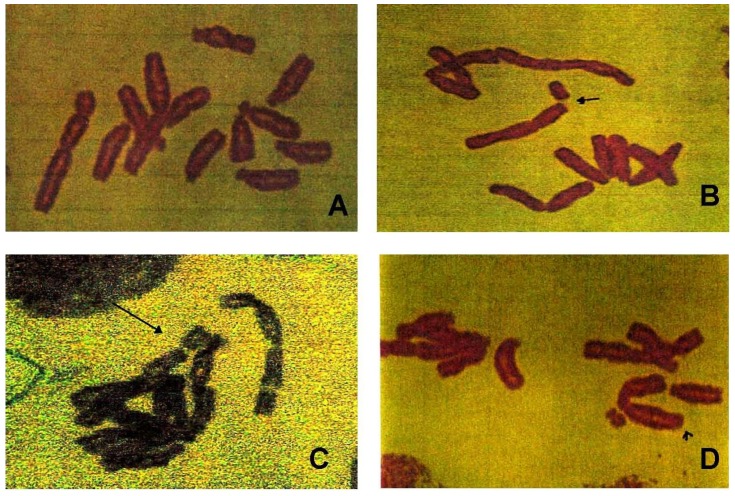
Representative images of metaphase chromosomes in root-tip meristem of *Vicia faba*. A = Normal chromosomes, B = break, C = Gap, D = deletion.

The metaphase analysis of *V. faba* root-tips revealed various types of chromosomal aberrations, which consisted of chromatid and isochromatid types of gaps, breaks, and fragments. A quantitative assessment of the distribution of breaks and gaps revealed that the distal regions of the chromosomes were more vulnerable to the effects of AgNPs. The results of the chromosomal aberration assay after treatment with AgNPs are summarized in Figure 4. The frequency of chromosomal aberrations (CA) also increased with increasing doses of AgNPs, and statistically significant differences (p < 0.05) from the control were observed. The mean percentages of the induced CAs were 4.2 ± 0.97%, 8.8 ± 0.95%, 13.6 ± 0.72%, 19.2 ± 1.58%, 39.7 ± 1.43% for 0, 12.5, 25, 50, 100 mg/L of AgNPs respectively. Representative aberrations are given in Figure 5(A,B).

### 3.4. Micronucleus Assay

**Figure 6:**
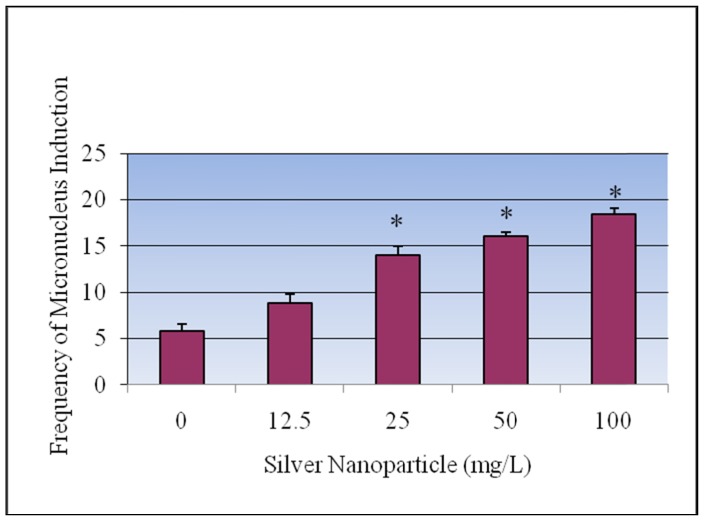
Effect of silver nanoparticles on the frequency of micronucleus induction in root-tip meristem of *Vicia faba*. Each experiment was done in triplicate. Data represents mean ± SD. Statistical significance (p < 0.05) is depicted as (*).

**Figure 7:**
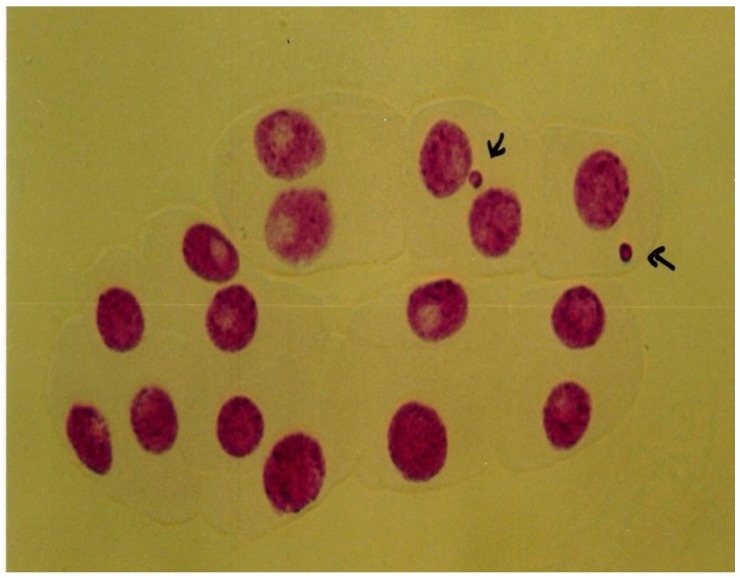
Representative images of micronuclei formation in the root-tip meristem of *Vicia faba.*

The results of the micronucleus assay of *V. faba* root-tip exposed to silver nanoparticles are summarized in Figure 6 and Figure 7. The frequency of micronuclei induction was dose-dependent and statistically significant differences (p < 0.05) from the control were observed. The frequencies of micronucleated cells were 5.86 ± 0.66, 8.87 ± 0.40, 14.06 ± 0.96, 16.06 ± 0.98, 18.4 ± 0.75 for 0, 12.5, 25, 50, 100 mg/L of AgNPs respectively. 

## 4. Discussion

Nanomaterials and nanoparticles have received considerable attention recently due to their unique properties and applications in diverse biotechnology and life science assays. Despite the rapid progress and early acceptance of nanobiotechnology, the potential for adverse effects in humans, non-humans biota and the ecosystems are yet to be thoroughly established. However, the environmental impact of nanomaterial is expected to increase substantially in the future. 

In the present investigation, we studied the clastogenic/genotoxic effects of AgNPs in *V. faba* root-tips using mitotic index (MI), structural chromosomal aberrations (CA) and micronuclei induction (MN) as the toxicological endpoints. We have noted a decrease in the mean mitotic index values in silver nanoparticle-exposed root-tips as compared to the controls. This could be due to a slower progression of cells from S (DNA synthesis) phase to M (mitosis) phase of the cell cycle as a result of AgNP exposure. It has been suggested that the cytotoxicity level can be determined by the decreased rate of the mitotic index [35]. Although it is most likely that this impairment in cell cycle progression is associated with silver nanoparticles toxicity, further experiments are needed to elucidate the biochemical mechanisms involved. At present, there are no published studies assessing the effect of silver nanoparticles on mitotic index in biological systems. As the mitotic index represents the number of dividing cells it accounts for the growth, and any decrease in mitotic index leads to the reduced growth. The reduction in mitotic index may be caused by the effect of the AgNP/test chemical on the microtubule [36,37].

Different types of structural chromosomal aberrations were observed with different concentrations of silver nanoparticle suspensions. The increase in the induction of structural chromosomal aberrations was found to be statistically significant in exposed root-tips compared to control. Out of all types of aberrations, chromatid breaks, isochromatid breaks, acentric fragments, minutes, translocations and gaps were the predominant forms of CA observed. These results are in agreement with the reports of [19,38]. Increases in percentage of aberrations in root meristems indicates genotoxic effects of test chemicals [35]. Number of factors can be contributing to the increased chromosomal aberrations. The most important one is due to the interference of chemicals during DNA repair. Different types of chromosomal aberrations by the chemicals/nanoparticles represent their clastogenicity. The chromosome gaps which involve only the loss of chromatin may be due to the loss of protein part of the chromosome [39]. The chromatid breaks, which represent the DNA double strand breaks that may not have undergone the G2 repair. Any such irreversible DNA damages will lead to the chromosomal aberrations. Irreversible DNA damage would be produced whenever the trapped cleavable complex collides with a replication fork, independently of whether it is euchromatic or heterochromatic regions of the chromosomes that are being replicate [40]. Root tips frequently used for cytogenetic studies in the past five decades were from *Allium cepa* and *Vicia faba* [31,33] which are excellent materials for clastogenicity studies of physical and chemical agents. 

Li *et al*. [41] and Chen *et al*. [42], while working with mammalian cell lines, demonstrated that the nanoparticles penetrated subcellular structures such as the mitochondria and nucleus causing uncoupling of respiration and increased oxidative stress. Tetramethylammonium hydroxide (TMA-OH) coated magnetic nanoparticles of ferrofluid induced chromosomal aberrations in the root meristem cells of *Zea mays* according to Racuciu *et al.* [43].

The results from this study showed that there was a statistically significant difference in the frequencies of MN induction in the *V. faba* root-tips exposed to AgNP when compared to control. Similar results were reported in *A. cepa* root-tips exposed to ZnO nanoparticles [38] and AgNP [44], showing an increase in the frequency of MN induction in a dose-dependent manner. Micronucleus formation is the result of acentric fragments or laggards being excluded from the nucleus proper during mitosis [32]. The increase in micronuclei also supports that the test chemicals are clastogenic and are capable of producing different types of chromosomal aberrations. Several hypotheses can be suggested to account for the clastogenic/genotoxic effects of silver nanoparticles, including the formation of adduct and/or damage at the level of DNA or chromosomes. DNA damaging agents have the potential to cause genomic instability, which is a predisposing factor in carcinogenesis. Hence, careful monitoring and further characterization of their systemic toxicity, genotoxicity and carcinogenicity is also essential.

## 5. Conclusions

Silver nanoparticles might penetrate plant systems and may interfere with intracellular components, impairing the stages of the cell division. In the present study a dose-dependent decrease in the MI was observed in the exposed group compare to control. There was an increase in the frequencies of CA and MN in root-tips of *V. faba*. Plant species are widely used for monitoring air pollution and for screening environmental chemicals for their genotoxic effects. The growing public debate on the toxicity and environmental impact of exposures to nanoparticles has not yet thoroughly established. Therefore it is imperative to determine a relatively inexpensive and commonly used short-term plant assay for *in situ* evaluation of the biological hazards of nanoparticles in the environment. The *V. faba* root tip chromosomal aberration assay is an established plant bioassay by IPCS to study such effects.
